# Design and cloning strategies for constructing shRNA expression vectors

**DOI:** 10.1186/1472-6750-6-1

**Published:** 2006-01-05

**Authors:** Glen J McIntyre, Gregory C Fanning

**Affiliations:** 1 Johnson and Johnson Research Pty Ltd, 1 Central Ave, Australian Technology Park, Eveleigh, NSW, 1430, Australia; 2 The school of Biological and Biomolecular Sciences, The University of New South Wales, Sydney, NSW 2052, Australia; 3 Tibotec BVBA, Gen De Wittelaan L 11 B3, 2800 Mechelen, Belgium

## Abstract

**Background:**

Short hairpin RNA (shRNA) encoded within an expression vector has proven an effective means of harnessing the RNA interference (RNAi) pathway in mammalian cells. A survey of the literature revealed that shRNA vector construction can be hindered by high mutation rates and the ensuing sequencing is often problematic. Current options for constructing shRNA vectors include the use of annealed complementary oligonucleotides (74 % of surveyed studies), a PCR approach using hairpin containing primers (22 %) and primer extension of hairpin templates (4 %).

**Results:**

We considered primer extension the most attractive method in terms of cost. However, in initial experiments we encountered a mutation frequency of 50 % compared to a reported 20 – 40 % for other strategies. By modifying the technique to be an isothermal reaction using the DNA polymerase Phi29, we reduced the error rate to 10 %, making primer extension the most efficient and cost-effective approach tested. We also found that inclusion of a restriction site in the loop could be exploited for confirming construct integrity by automated sequencing, while maintaining intended gene suppression.

**Conclusion:**

In this study we detail simple improvements for constructing and sequencing shRNA that overcome current limitations. We also compare the advantages of our solutions against proposed alternatives. Our technical modifications will be of tangible benefit to researchers looking for a more efficient and reliable shRNA construction process.

## Background

**Figure 1:**
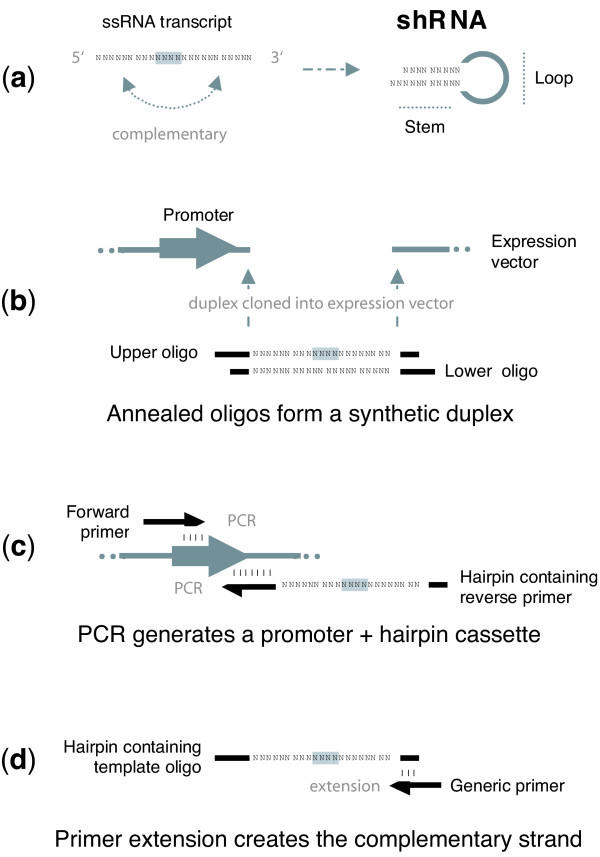
*Design strategies for creating short hairpin RNA (shRNA) template inserts*. (**a**) Expressed shRNA is transcribed as a ssRNA molecule that folds onto itself forming a stem-loop structure. (**b**) **Annealed complementary oligos **can be used to create a synthetic DNA duplex (74 % of studies) for cloning. (**c**) Similar inserts for cloning can be made as complete promoter-shRNA cassettes by **PCR **using a hairpin containing primer (22 % of studies). (**d**) Alternatively, **extension **of a generic primer bound to a unique 'template' oligo can create a synthetic insert, akin to the annealed complementary oligo strategy (4 % of studies).

RNA interference (RNAi) is the pathway by which short interfering RNA (siRNA) or short hairpin RNA (shRNA) are used to inactivate the expression of target genes (for recent review see [1,2]). Compared to siRNA, shRNA offers advantages in silencing longevity, delivery options and cost. Expressed shRNA is transcribed in cells from a DNA template as a single-stranded RNA molecule (~50 – 100 bases) (Fig. 1a). Complementary regions spaced by a small 'loop' cause the transcript to fold back on itself forming a 'short hairpin' in a manner analogous to natural microRNA. Recognition and processing by the RNAi machinery converts the shRNA into the corresponding siRNA. In a survey of more than 100 papers applying expressed shRNA in mammalian systems we determined that shRNA expression vectors are constructed by one of three different methods (see Additional file 1).

The most common method for making shRNA constructs (74 % of surveyed studies) requires the synthesis, annealing and ligation of two complementary oligonucleotides (oligos) into an expression vector (Fig. 1b and Additional file 2). While this cloning method is quick, the oligo synthesis cost is nearly double that of other methods and the frequency of false positives determined by sequencing is high (typically 20 – 40 %) [3]. The unreliability of this method is in part due to the difficulty in the synthesis of long oligos (length > 35 bases) [4]. As this method requires two long oligos the chance of mutation due to synthesis error is doubled.

The second strategy (employed in 22 % of studies) is a PCR approach in which a promoter sequence serves as the template (Fig. 1c). The hairpin sequence is contained in the reverse primer and PCR results in a cloning cassette comprising both promoter and hairpin. Correct amplicon production is critically dependent upon the sequence of the reverse primer. Hence this technique requires costly purification (e.g. PAGE) of the long reverse primer to exclude truncated oligos originating from the manufacturing process [5]. Although it is advantageous that only a single long oligo is required, the strong secondary structure predicted to form within this primer can lead to the amplification of false products. To alleviate these problems, the long reverse primer can be exchanged for two shorter primers. The reaction is then modified to a two-round nested PCR with each primer introducing half the hairpin per round [6]. However, the repeated cycling inadvertently increases the chance of incorporating polymerase-induced mutations.

The third method (applied in 4 % of studies) encompasses several techniques relating to primer extension. Each is based on the principle of a polymerase extending the 3' end of overlapping oligos [7]. In one instance the shRNA template is formed from two long partially complementary oligos of approximately equal length, overlapping at their 3' ends [8,9]. Each oligo serves as both template (for extending the opposite oligo) and primer (to copy the opposite oligo). Extension and repeated cycling generates a double-stranded product, akin to that generated in the annealed oligo method. In a variation of this method, one long oligo is used as the template and a second short oligo (generic) is used as the primer for extension (Fig. 1d and Additional file 2). The product can be further amplified by PCR with addition of another short primer binding the extended strand [4]. This technique is the cheapest of all the construction methods discussed as it both halves the cost of unique oligos (compared to the annealed oligo method) and does not need costly oligo purification (compared to the promoter based PCR method). However, this saving may be off-set by a high rate of polymerase-induced mutation in either the initial extension step or by repeated cycling [4].

A common drawback of constructing shRNA vectors, irrespective of the method used, is the difficulty in confirming the sequence of the hairpin region using automated sequencing protocols. It has been widely reported that hairpin templates can lead to sequencing reactions that terminate prematurely, at sites adjacent to or just within the region that encodes the hairpin stem [3,10-12] (personal observations). Although this phenomenon is commonly encountered, it does not affect all hairpins equally and is very likely dependent on the strength of secondary structures that are unique to each sequence.

The purpose of this study, therefore, was to determine the most cost effective and reliable method for producing multiple shRNA expression constructs. Two parameters were tested: (**i**) cloning strategies and (**ii**) methodologies to ensure that all hairpin templates could be confirmed at high-throughput automated sequencing facilities.

## Results and Discussion

**Figure 2:**
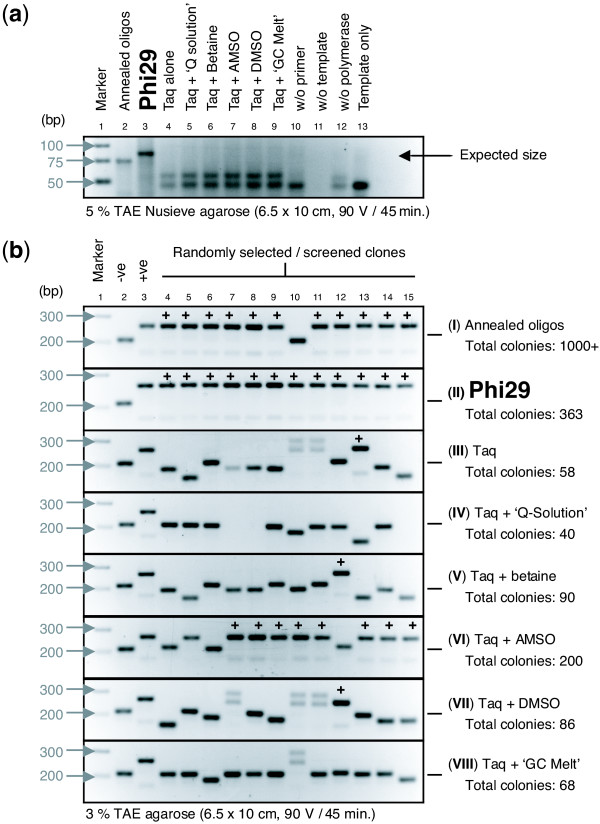
*Phi29 DNA polymerase can be used for highly efficient shRNA template generation*. Taq likely encounters difficulty when attempting to read through highly structured templates such as shRNA, despite the inclusion of additives designed to overcome secondary structure. (**a**) The extension reaction using Phi29 is the only reaction to generate detectable full-length double-stranded product comparable to the annealed complementary oligos. (**b**) Screening via colony PCR (across the insertion point) reveals that out of all the extensions reactions, the one using phi29 DNA polymerase yields the greatest number of colonies (Total colonies) and recombinant clones (+).

Upon consideration of the available methods for shRNA construction, we selected primer extension using a long template oligo and a short universal primer as it was the least costly. Our first step to reduce mutations was to remove the possibility of cycling-induced errors by conducting all reactions as single-step extensions. Even so, our initial experiments using Taq polymerase were not encouraging as, (**i**) the total number of colonies recovered was low (Fig. 2), (**ii**) of these only a small fraction contained a correctly sized insert (often less than 10 %) and (**iii**) upon sequencing it was found that up to 50 % of these 'positive' recombinant clones contained substitutions and deletions. The low recovery rate and high incidence of mutations were most likely due to the predicted secondary structure of the hairpin template [13] inhibiting Taq polymerase chain elongation. The extensive end-stage screening and sequencing of bacterial clones made this protocol, as it stood, impractical. To address these shortcomings, we tested a panel of molecular disruption agents to reduce secondary structure formation during chain elongation. Agents tested included; Q-solution (1×, Qiagen), betaine (1 M), ammonium sulfate (AMSO, 15 mM), dimethyl sulfoxide (DMSO, 5 %) and GC-Melt (1 M, BD Biosciences). None of the additives tested yielded a detectable full-length extension product (Fig. 2a), although the addition of AMSO did improve on the number of recombinant clones (Fig. 2b). There was, therefore, little improvement over protocols employing Taq alone.

To improve upon these results, we substituted Taq polymerase with an enzyme better able to counter the secondary structure of the hairpin template. Phi29 is an enzyme that facilitates rolling circle replication by the *Bacillus subtilis *phage Φ29 [14], and as such possesses strand displacing capabilities [15]. In addition, the supplier's comparison of fifteen available polymerases suggested that Phi29 possessed the highest displacing activity (New England Biolabs). On testing Phi29 we found it was able to copy a highly structured template oligo, yielding detectable full-length product (Fig. 2a). This resulted in higher cloning efficiencies (Fig. 2b) and a lower mutation rate (10 %) when compared to Taq (50 %) (Table 1). The mutation frequency was even lower than that reported for the annealed oligo cloning strategy (20 – 40 %) [3]. Furthermore, with a nucleotide polymerization rate ranging from 290 nt./min. @ 4°C to 2280 nt./min. @ 30°C [16] the reaction is fast, isothermal and independent of additives. We also confirm the previous finding that oligos for primer extension need only be ordered at the minimal synthesis and purification scales (0.05 μM, desalt) [4].

The use of another enzyme with similar properties, Vent, has also been reported [4]. However, additives (DMSO and GC-Melt) and repeated thermocycling were recommended for successful extension. Whilst valid, this technique was hampered by the occurrence of cycling-induced errors. In summary, our isothermal procedure using Phi29 retains the cost benefits of primer extension and reduces manifestations of both synthesis and polymerase-induced mutations.

**Figure 3:**
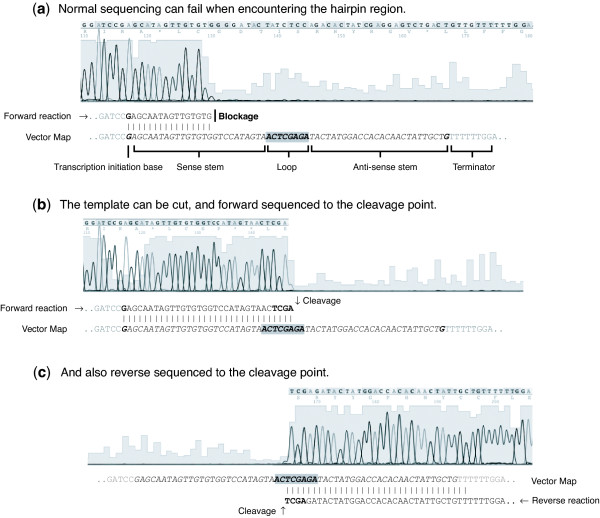
*Loop digestion can be used to successfully determine the sequence of hairpin templates that are refractory to ordinary sequencing techniques*. (**a**) Strong secondary structure predicted to form in the vector template used for sequencing can 'block' chain elongation, thus terminating the reaction. (**b**) This can be overcome by first digesting the template within the loop encoding region and sequencing half the template from the forward direction, and (**c**) the other half from the reverse direction. A small degree of sequence overlap between the forward and reverse reactions, at the restriction site (shown in bold), ensures that every position of the template can be verified.

During this study, we generated multiple shRNA expression constructs; all of which required sequence confirmation. Given the prevalence of mutations, this step becomes imperative as suppressive activity is dependent on homology between the siRNA guide strand and target RNA [17,18]. Unfortunately, sequencing shRNA constructs is not always straightforward [3,10-12]. We often found that the standard sequencing procedure failed, again most likely due to the inability of the polymerase to read-through the highly structured template (Fig. 3a). Neither repositioning the sequencing primer, nor the addition of molecular disruption agents to the reaction were able to overcome sequencing limitations (**data not shown**). Although our work with Phi29 suggests an obvious solution, it was not possible to exchange the sequencing polymerase when using automated sequencing facilities.

As an alternative, we found that inclusion of a unique restriction enzyme (RE) site within the loop sequence allows the vector to be linearised and sequenced in two separate reactions; one for the sense and one for the anti-sense (Fig. 3). Our present design incorporates a centrally located *Xho*I site in an 8 base loop (A**CTCGAG**A), but it is probable that other RE sites could also be employed. We found that the digestion could be performed directly in the sample tube destined for sequencing, with no impact on sequencing quality (see **Methods **for details). From our survey we also noted that although uncommon, the inclusion of an RE site within the hairpin loop was not unique (used in 8 % of cases), but its only described use was to assist in screening and selection of recombinant clones [12]. In no case was there a reported link, as we propose, between RE loop design and the benefits of dual-sequencing the digested vector.

**Figure 4:**
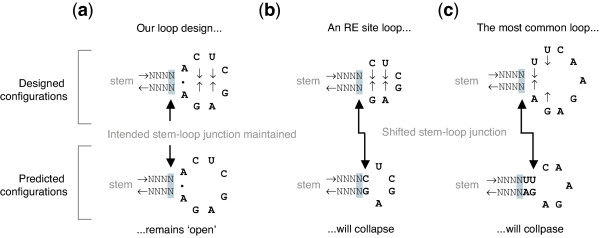
*Internal base pairing can cause intended loop configurations to collapse*. (**a**) Structural predictions reveal that an 8 base loop containing a 6 base restriction site (**A**CUCGAG**A**) will remain in an 'open' configuration by virtue of a mismatched nucleotide pair positioned adjacent to the end of the stem. (**b**) The equivalent 6 base loop (**C**UCGA**G**), without a mismatched pair, is predicted to partially collapse, shortening the open loop to 4 bases and simultaneously extending the stem length (by 1 bp) thus altering the position of the stem-loop junction. (**c**) A commonly employed 9 base loop (**UU**CAAGA**GA**, 60 % of studies) is also predicted to collapse, forming a 5 base open loop with a similarly shifted stem-loop junction.

Our design incorporates an additional mismatched nucleotide pair placed adjacent to the end of the stem (**A**CUCGAG**A**, mismatches indicated in bold). Structural predictions reveal this to be a necessary inclusion to ensure that the loop, based on a palindromic RE site, remains in an open configuration (Fig. 4). This is important as additional paired nucleotides at the base of the loop effectively increase stem length, shifting the intended stem-loop junction. It has been demonstrated, for analogous microRNA structures, that altering the stem-loop junction has possible consequences for ensuing cleavage, processing, target recognition and hence suppressive activity [19] – an observation that we have also noted for shRNA molecules (**manuscript in preparation**). Surprisingly, 60 % of surveyed studies employed the loop sequence, **UU**CAAGA**GA**, which *is *predicted to internally pair (**UU**.. to ..**GA**), potentially altering suppressive activity as described (Fig. 4). The loop design we propose is amenable to any hairpin sequence without altering the internal stem, stem-loop junction or consequent siRNA characteristics.

**Figure 5:**
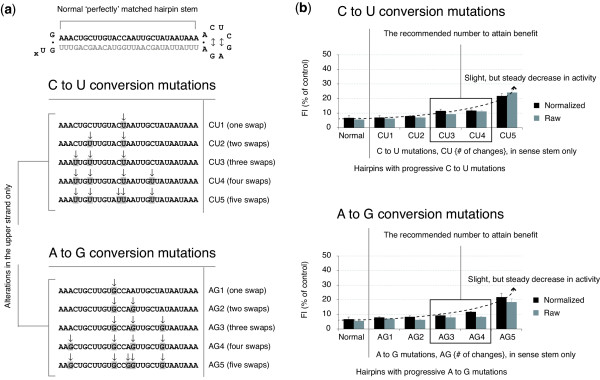
*Hairpins with mismatched or bulged bases in the sense stem are less potent gene suppressors*. (**a**) Introduced mutations were either changes of a C residue to a **U **residue (from 1 to 5) or an A residue to a **G **residue (from 1 to 5) and were compared against the normal (perfectly matched) shRNA with no mutations ("Normal"). All alterations were in the sense strand only (designed to give rise to the non-active siRNA 'passenger' strand), whilst the anti-sense strand of the shRNA stem (designed to give rise to the active siRNA 'guide' strand) remained the same, and perfectly complementary to the target in all variations. (**b**) Reduction in suppressive activity (relative to the perfectly matched shRNA) was minimal for 1 to 2 mismatched bases but more notable for the constructs with the recommended 3 to 4 mismatched bases, and most obvious where the mutation is increased to 5 bases.

Another reported strategy to alleviate sequencing difficulties is to include mismatched bases within the shRNA stem [3,11]. Additionally, it has been proposed that this also reduces the occurrence of bacterially-derived mutation events. The mismatches are positioned such that the anti-sense stem (designed to be the siRNA 'guide' strand) is complementary to the target but mismatched to the sense stem (suggested as 3 or 4 'C to U', or 'A to G' conversions). We attempted this using the annealed oligo strategy yet still observed an ~27 % mutation rate – a figure comparable to fully complementary stem designs [3]. While we did see a reduction in sequencing difficulties when mismatches were present, we also observed a correlation between increasing mismatches and decreasing gene suppression activity (Fig. 5). We can only speculate that these disparities with the original observations were due to sequence-specific effects (resulting in activity differences) or different bacterial lab strains (resulting in mutation differences). With reference to the latter, commonly used *E.coli *strains such as DH5α encode sbcC and sbcD, which are proteins known to generate double-stranded breaks in DNA hairpins [20]. We have found that engineered *sbcCD *deletion strains such as GT116 (Invivogen), specifically developed to tolerate inverted repeat regions in DNA, yield more faithful recombinant clones.

It is worthy of final note that we see no obvious correlation in our data between hairpin stem length (having generated lengths from 15 – 45 bp) and the incidence of mutations arising during cloning or problems with sequencing. In our hands they appear largely sequence dependent as we encountered long and short hairpins that were problematic on both counts.

## Conclusion

We have analyzed the literature and determined that shRNA construction is frequently associated with difficulties and can be hindered by high mutation frequencies in accordance with our own observations. Our investigations to find an improved alternative led to a variation of the primer extension method using Phi29. The procedure is swift, isothermal and independent of additives making it, in our hands, the most reliable and cost effective of all the construction techniques. In addition, we present a simple and robust solution for overcoming sequencing limitations commonly encountered with shRNA vectors. This solution is based on an RE loop design, which is amenable to any shRNA without compromising its suppressive activity. These technical modifications will be of tangible benefit to researchers looking to improve their shRNA construction process.

## Methods

### shRNA template generation using complementary annealed oligonucleotides

Our expression vector (derived from pSILENCER-3.0H1, Ambion) contained a human H1 polymerase-III (pol-III) promoter for shRNA expression. Each shRNA insert was designed as a synthetic duplex with overhanging ends identical to those created by restriction enzyme (RE) digestion (*Bam*HI at the 5' and *Hind*III at the 3') (see Additional file 2 for diagram). The coding region for each hairpin was contained within a single oligonucleotide (upper oligo: 5'-**GATCC **[G/A]N_(19–29)_A**CTCGAG**AN_(19–29) _[G/A/C]TTTTTTGG**A**-3') and its complementary equivalent (lower oligo: 5'-**AGCTT**CCAAAAAA [G/A/C]N_(19–29)_A**CTCGAG**AN_(19–29) _[G/A]**G**-3'). These ranged in size from 60 – 100 bases (for hairpins with 19 – 29 bp stems). Each duplex contained a transcription initiation base (if required), the shRNA encoding region (sense stem, loop sequence and anti-sense stem), a termination spacer (if required) and a pol-III termination signal consisting of a run of at least 4 'T's. The transcription initiation base was an 'A' or 'G' (required for efficient pol-III transcription initiation) and was only included if the first base of the hairpin stem was not a purine. The termination spacer was any base but 'T' and was included only if the last base of the anti-sense stem was 'T' so as to prevent premature termination via an early run of 'T's. Oligos were ordered at the minimal synthesis and purification scales (0.05 μM and desalt, Sigma-Genosys). Each oligo was re-suspended in water (1 – 10 μg/μl) and 1 μl from each was added to 98 μl of annealing solution (10 mM Tris pH 8.0, 50 mM NaCl, 1 mM EDTA), heated to 100°C for 5 minutes, slowly equilibrated to room temperature and diluted up to 10,000 fold for ligation. The insert and vector were ligated, and used to transform electrocompetent GT116 *E.coli *(Invivogen). Positive clones were confirmed by automated sequencing using our loop digestion method.

### shRNA template generation using Phi29 primer extension

Each template oligo was similar in design to the upper oligo of the annealed oligo method (5'-GCGC**GGATCC**[G/A]N_(19–29)_A**CTCGAG**AN_(19–29)_[G/A/C]TTTTTTGG**AAGCTT**-3') but the ends were extended to encode the entire sequence of the RE sites plus an additional 5' 'seat' sequence to facilitate RE binding and digestion of the extended product (see Additional file 2 for diagram). A short primer was designed to bind at the 3' end of the template oligo and introduced the 3' seat (5'-CGCG**AAGCTT**CCAAAAAA-3'). Both template and primer oligos were synthesized and re-suspended as previously described for the annealed oligo method. Twenty picomoles of each oligo was used in the extension reaction (1× reaction buffer, 2× BSA, 50 mM final conentration of dNTPs, 10 units of Phi29 (New England Biolabs) and water to 20 μl), which was incubated at 30°C for ~10 min., then 65°C for 10 min. (to disable the polymerase). The extension product was digested (*Bam*HI plus *Hind*III), purified using the Nucleotide Removal kit (Qiagen), ligated to the expression vector and used to transform electrocompetent GT116 *E.coli*. Positive clones were confirmed by automated sequencing using our loop digestion method.

### shRNA sequence confirmation by loop digestion

Each construct was digested and sequenced in two reactions, one containing the forward primer, the other containing the reverse. The primers bound to the expression vector backbone approximately 100 bases away from the region encoding the base of the hairpin stem. Each reaction contained: 1× RE buffer (NEB-2, New England Biolabs), 1× BSA, ~500 ng of template vector, 10 pmol of sequencing primer, ~10 units *Xho*I and water to a total volume of 16 μl, and was incubated at 37°C for 30 – 60 min. prior to shipping, without purification, to an automated sequencing facility (Australian Genome Research Facility, AGRF).

### shRNA suppressive activity assay

Suppressive activity was determined by transient triple-transfection of the expression vector (H1 driving expression of the shRNA) with a matched GFP-target fusion assay vector (color 1) and a normalization control vector (color 2) into an adherent mammalian cell line (HEK293a). Fluorescence levels were determined by Flow Cytometry analysis 48 hours post-transfection. Values are given in Fluorescence Index units (FI; obtained by dividing the average geo mean fluorescence by the total number of cells within the analysis gate) both normalized to the second color (to account for non-specific effects) and raw (to show the extent of non-specific effects) and presented as a percentage of the FI for the expression vector control (obtained when co-transfecting the empty – non-hairpin containing – expression vector). Each data column represents the average of 3 replicated samples with 95 % confidence intervals shown.

## Authors' contributions

G. J. M. conceived and performed the experiments. G. C. F. participated in the experimental conception and the assessment of results. Both authors read and approved the final manuscript.

## Supplementary Material

Additional File 1 *A survey of studies that employed expressed shRNA revealed that all shRNA constructs are made from one of three possible methods*. A random selection of published studies using expressed shRNA were surveyed and scored for their method of shRNA construction which could be classified as one of three different strategies (see main text for detailed descriptions); (**i**) Annealed complementary oligonucleotides, (**ii**) Promoter based PCR or (**iii**) Primer extension. Click here for file

Additional File 2 *Diagrams for shRNA template generation via complementary annealed oligonucleotides or primer extension using Phi29 DNA polymerase*. Both the most common shRNA insert construction technique (complementary annealed oligos) and our proposed alternative (primer extension using Phi29) are diagrammatically represented indicating oligo alignments and the features of each oligo. Click here for file

## Figures and Tables

**Table 1:** *Sequence mutations are more commonly encountered when constructing shRNA templates by primer extension using Taq rather than Phi29*. A representative subset of sequencing results for a selection of constructs that were made twice, once using Taq and once using Phi29. Constructs generated by Taq were more prone to mutations than when made with Phi29. Both substitutions and deletions were commonly encountered. These were positioned in either the stem or the loop encoding regions. Templates generated by Phi29 were not refractory to mutations (*); however, the frequency and extent of mutation was below that observed for Taq (in this case we found 50 % (8/16) of Taq generated constructs contained mutations versus 10 % of similar constructs made with Phi29).

**ShRNA**	**Stem Length**	**Mutations when using Taq (location)**	**Mutations when using Phi29 (location)**
**A**	18 bp	**1 **deletion (stem)	-
**B**	21 bp	**1 **deletion (loop), **8 **substitutions (stem)	-
**C**	21 bp	**2 **substitutions (loop)	-
D *	22 bp	**1 **deletion (stem), **2 **substitutions (stem)	**1 **substitution (stem)
**E**	25 bp	**1 **deletion (stem)	-
F *	25 bp	**3 **deletions (stem)	**1 **deletion (stem)
**G**	27 bp	**1 **deletion (stem)	-
