# Supplementary material for: Design and cloning strategies for constructing shRNA expression vectors
Source: BMC Biotechnol. 2006 Jan 5;6:1. doi: 10.1186/1472-6750-6-1 (PMC1343552; doi:10.1186/1472-6750-6-1)
Supplement: Additional File 1 — A survey of studies that employed expressed shRNA revealed that all shRNA constructs are made from one of three possible methods. A random selection of published studies using expressed shRNA were surveyed and scored for their method of shRNA construction which could be classified as one of three different strategies (see main text for detailed descriptions); (i) Annealed complementary oligonucleotides, (ii) Promoter based PCR or (iii) Primer extension. [file 1472-6750-6-1-S1.pdf]

Additional file **1**

A survey of studies that employed expressed shRNA revealed that all shRNA constructs are made from one of three possible methods

A random selection of over 100 published studies using expressed shRNA were surveyed for their method of shRNA construction, which could be classified as one of three different strategies (see main text for detailed descriptions); (**i**) **Annealed** complementary oligonucleotides (oligos) (87 cases): complementary oligos were annealed in pairs to create a synthetic DNA duplex. (**ii**) Promoter based **PCR** (26 cases): cloning cassettes containing a promoter and the hairpin encoding region were made by PCR. (**iii**) Primer **extension** (4 cases): the shRNA template is formed from two partially complementary oligos, overlapping at their 3' ends, or in a variation of this method one long oligo is used as the template and a second short oligo is used as a primer for extension.

**Reference:**	**Method:**
J. Anderson, R. Akkina, *AIDS Res Ther* **2**, 1 (Jan 13, 2005).	PCR*
J. F. Arrighi *et al.*, *J Virol* **78**, 10848 (Oct, 2004).	Annealed
A. M. Babcock *et al.*, *Mol Ther* **11**, 899 (Jun, 2005).	Annealed
A. Bahi, F. Boyer, M. Kolira, J. L. Dreyer, *J Neurochem* **92**, 1243 (Mar, 2005).	PCR
D. Bernard *et al.*, *Oncogene* (May 9, 2005).	Annealed*
K. Berns *et al.*, *Nature* **428**, 431 (Mar 25, 2004).	Annealed
D. Boden, O. Pusch, F. Lee, L. Tucker, B. Ramratnam, *Mol Ther* **9**, 396 (Mar, 2004).	Annealed
D. Boden *et al.*, *Nucleic Acids Res* **32**, 1154 (2004).	Annealed
I. Bot *et al.*, *Blood* (May 10, 2005).	Annealed
H. Caldas, M. P. Holloway, B. M. Hall, S. J. Qualman, R. A. Altura, *J Med Genet* (May 20, 2005).	Annealed*
H. B. Cao *et al.*, *Cell Res* **15**, 111 (Feb, 2005).	Annealed
W. Cao, R. Hunter, D. Strnatka, C. A. McQueen, R. P. Erickson, *J Appl Genet* **46**, 217 (2005).	Annealed
S. M. Cashman, E. A. Binkley, R. Kumar-Singh, *Gene Ther* (May 5, 2005).	Annealed
D. Castanotto, L. Scherer, *Methods Enzymol* **392**, 173 (2005).	Annealed
H. M. Chan, M. Narita, S. W. Lowe, D. M. Livingston, *Genes Dev* **19**, 196 (Jan 15, 2005).	Annealed*
L. J. Chang, X. Liu, J. He, *Gene Ther* (Mar 3, 2005).	Annealed
L. M. Chen *et al.*, *World J Gastroenterol* **11**, 831 (Feb 14, 2005).	Annealed
T. J. Corydon, J. Hansen, P. Bross, T. G. Jensen, *Mol Genet Metab* (May 28, 2005).	Annealed
J. Cummings *et al.*, *Br J Cancer* **92**, 532 (Feb 14, 2005).	PCR
A. T. Das *et al.*, *J Virol* **78**, 2601 (Mar, 2004).	Annealed
B. L. Davidson, S. Q. Harper, *Methods Enzymol* **392**, 145 (2005).	PCR
M. A. Denti, A. Rosa, O. Sthandier, F. G. De Angelis, I. Bozzoni, *Mol Ther* **10**, 191 (Jul, 2004).	Annealed
Y. Einav, R. Agami, D. Canaani, *FEBS Lett* **579**, 199 (Jan 3, 2005).	Annealed
R. J. Fish, E. K. Kruithof, *BMC Mol Biol* **5**, 9 (Aug 3, 2004).	PCR
S. Franco *et al.*, *Blood* **104**, 3927 (Dec 15, 2004).	Annealed*
H. Fung, B. Demple, *Mol Cell* **17**, 463 (Feb 4, 2005).	Annealed*
L. Gewin, H. Myers, T. Kiyono, D. A. Galloway, *Genes Dev* **18**, 2269 (Sep 15, 2004).	PCR
S. Gonzalez *et al.*, *Mol Ther* **11**, 811 (May, 2005).	PCR
D. Grimm, K. Pandey, M. A. Kay, *Methods Enzymol* **392**, 381 (2005).	Annealed
Y. Guo *et al.*, *World J Gastroenterol* **11**, 2912 (May 21, 2005).	Annealed
S. Gupta, R. A. Schoer, J. E. Egan, G. J. Hannon, V. Mittal, *Proc Natl Acad Sci U S A* **101**, 1927 (2004).	Annealed
D. L. Hacker, M. Bertschinger, L. Baldi, F. M. Wurm, *Gene* **341**, 227 (Oct 27, 2004).	Annealed
G. Hernandez-Hoyos, J. Alberola-Ila, *Methods Enzymol* **392**, 199 (2005).	PCR

I. Hernandez-Munoz *et al.*, *Proc Natl Acad Sci U S A* **102**, 7635 (May 24, 2005).	Annealed*
C. C. Ho *et al.*, *Exp Cell Res* **304**, 1 (Mar 10, 2005).	Annealed
T. Hosono *et al.*, *Gene* **348**, 157 (Mar 28, 2005).	Annealed
J. Hua, D. G. Mutch, T. J. Herzog, *Gynecol Oncol* (May 24, 2005).	Annealed
A. Huang *et al.*, *FEBS Lett* **558**, 69 (Jan 30, 2004).	Annealed*
B. Huang, S. Kochanek, *Hum Gene Ther* **16**, 618 (May, 2005).	Annealed
L. Hung, V. Kumar, *FEBS Lett* **560**, 210 (Feb 27, 2004).	Annealed
C. Hurtado *et al.*, *J Mol Cell Cardiol* **38**, 647 (Apr, 2005).	PCR
K. Izeradjene, L. Douglas, A. Delaney, J. A. Houghton, *Oncogene* **24**, 2050 (Mar 17, 2005).	Annealed*
A. C. Jenke *et al.*, *Hum Gene Ther* **16**, 533 (Apr, 2005).	PCR
M. C. Kamradt *et al.*, *J Biol Chem* **280**, 11059 (Mar 25, 2005).	Annealed
T. Kanda, B. Zhang, Y. Kusov, O. Yokosuka, V. Gauss-Muller, *Biochem Biophy Res Com* **330**,1217(2005).	Annealed
T. Kudo, S. Sutou, *J Reprod Dev* (Apr 5, 2005).	Annealed
D. Kuninger *et al.*, *Hum Gene Ther* **15**, 1287 (Dec, 2004).	Annealed
A. Laatsch, S. Ragozin, T. Grewal, U. Beisiegel, H. Joerg, *Eur J Cell Biol* **83**, 113 (Apr, 2004).	Annealed
S. S. Lakka *et al.*, *Oncogene* **23**, 4681 (Jun 10, 2004).	Annealed
L. S. Lambeth *et al.*, *BMC Biotechnol* **5**, 13 (May 11, 2005).	PCR
M. A. Langlois *et al.*, *J Biol Chem* **280**, 16949 (Apr 29, 2005).	PCR
S. K. Lee *et al.*, *Blood* (Apr 14, 2005).	Annealed
F. Leenders *et al.*, *Embo J* **23**, 3303 (Aug 18, 2004).	Annealed
S. Lefrancois, M. Canuel, J. Zeng, C. R. Morales, *Biol Proced Online* **7**, 17 (2005).	Annealed*
M. J. Li, R. McMahon, D. S. Snyder, J. K. Yee, J. J. Rossi, *Oligonucleotides* **13**, 401 (2003).	PCR
M. J. Li, J. J. Rossi, H. Le Hir, A. Nott, M. J. Moore, *Methods Enzymol* **392**, 218 (2005).	Annealed
S. L. Li, R. S. Dwarakanath, Q. Cai, L. Lanting, R. Natarajan, *J Lipid Res* **46**, 220 (Feb, 2005).	PCR
X. P. Li, G. Li, Y. Peng, H. F. Kung, M. C. Lin, *Biochem Biophys Res Commun* **315**, 212 (Feb 27, 2004).	PCR
Z. Li *et al.*, *FEBS Lett* (May 21, 2005).	Annealed
X. Lin *et al.*, *FEBS Lett* **577**, 376 (Nov 19, 2004).	PCR
X. Ling, R. B. Arlinghaus, *Cancer Res* **65**, 2532 (Apr 1, 2005).	Annealed*
X. D. Liu, S. M. Ma, Y. Liu, S. Z. Liu, A. Sehon, *Biochem Biophys Res Commun* **324**, 1173 (2004).	Annealed
A. Lu *et al.*, *Virology* **324**, 84 (Jun 20, 2004).	Annealed
W. W. Lu, Y. Y. Hsu, J. Y. Yang, S. H. Kung, *Biochem Biophys Res Commun* **325**, 494 (Dec 10, 2004).	Annealed
Y. Matthess *et al.*, *Oncogene* **24**, 2973 (Apr 21, 2005).	Annealed
D. C. McManus *et al.*, *Oncogene* **23**, 8105 (Oct 21, 2004).	PCR
V. M. Miller, C. M. Gouvion, B. L. Davidson, H. L. Paulson, *Nucleic Acids Res* **32**, 661 (2004).	PCR
S. Miyagawa *et al.*, *J Biochem (Tokyo)* **137**, 503 (Apr, 2005).	Annealed
M. Miyagishi, H. Sumimoto, H. Miyoshi, Y. Kawakami, K. Taira, *J Gene Med* **6**, 715 (Jul, 2004).	Annealed
S. Modem, K. R. Badri, T. C. Holland, T. R. Reddy, *Nucleic Acids Res* **33**, 873 (2005).	Annealed
M. D. Moore, M. J. McGarvey, R. A. Russell, B. R. Cullen, M. O. McClure, *J Gene Med* (Mar 9, 2005).	Annealed*
P. Nahreini, A. J. Hanson, C. P. Andreatta, W. T. Koustas, K. N. Prasad, *Cell Mol Neurobio* **24**, 781(2004).	Annealed
L. J. Nicholson, M. Philippe, A. J. Paine, D. A. Mann, C. T. Dolphin, *Mol Ther* **11**, 638 (Apr, 2005).	Annealed
P. J. Paddison *et al.*, *Nat Methods* **1**, 163 (2004).	Extension^
P. J. Paddison *et al.*, *Nature* **428**, 427 (Mar 25, 2004).	PCR
C. P. Paul, *Methods Enzymol* **392**, 125 (2005).	Annealed
S. Pebernard, R. D. Iggo, *Differentiation* **72**, 103 (Mar, 2004).	PCR
R. Piva *et al.*, *Blood* **105**, 1750 (Feb 15, 2005).	Annealed
V. Poltoratsky, J. K. Horton, R. Prasad, S. H. Wilson, *DNA Repair (Amst)* (Jun 8, 2005).	Annealed
T. Pruss, M. Niere, E. U. Kranz, H. Volkmer, *Eur J Neurosci* **20**, 3184 (Dec, 2004).	Annealed
X. R. Ren, L. J. Zhou, G. B. Luo, B. Lin, A. Xu, *J Viral Hepat* **12**, 236 (May, 2005).	Annealed

S. Rotolo *et al.*, *Int J Cancer* **115**, 164 (May 20, 2005).	Annealed
L. J. Scherer *et al.*, *Mol Ther* **10**, 597 (Sep, 2004).	PCR
M. Scherr, K. Battmer, B. Schultheis, A. Ganser, M. Eder, *Gene Ther* **12**, 12 (Jan, 2005).	Annealed
J. Seibler *et al.*, *Nucleic Acids Res* **33**, e67 (2005).	PCR
R. G. Shah, M. M. Ghodgaonkar, B. Affar el, G. M. Shah, *Biochem Biophys Res Commun* **331**, 167 (2005).	Annealed**
J. Song, S. Pang, Y. Lu, R. Chiu, *Biochem Biophys Res Commun* **323**, 573 (Oct 15, 2004).	Annealed
B. Spankuch *et al.*, *J Natl Cancer Inst* **96**, 862 (Jun 2, 2004).	Annealed
Y. Takabatake *et al.*, *Gene Ther* **12**, 965 (Jun, 2005).	Annealed
Y. Takigawa *et al.*, *Microbiol Immunol* **48**, 591 (2004).	Annealed
M. Taniai *et al.*, *Cancer Res* **64**, 3517 (May 15, 2004).	Annealed
P. Tao *et al.*, *Chin Med J (Engl)* **118**, 714 (May 5, 2005).	Annealed*
M. Tatsuka *et al.*, *Oncogene* **24**, 1122 (Feb 3, 2005).	Annealed
G. Tiscornia, V. Tergaonkar, F. Galimi, I. M. Verma, *Proc Natl Acad Sci U S A* **101**, 7347 (May 11, 2004).	Annealed*
H. J. Unwalla *et al.*, *Nat Biotechnol* (Nov 28, 2004).	Extension^
A. Ventura *et al.*, *Proc Natl Acad Sci U S A* **101**, 10380 (Jul 13, 2004).	Annealed
M. Wakiyama, T. Matsumoto, S. Yokoyama, *Biochem Biophys Res Commun* **331**, 1163 (Jun 17, 2005).	PCR
X. Wang *et al.*, *Int J Cancer* **112**, 994 (Dec 20, 2004).	Annealed
C. Weiss-Haljiti *et al.*, *J Biol Chem* **279**, 43273 (Oct 8, 2004).	PCR
M. T. Wu *et al.*, *Biochem Biophys Res Commun* **330**, 53 (Apr 29, 2005).	Annealed
X. Xu, P. Shrager, *J Neurosci Res* **79**, 428 (Feb 15, 2005).	Annealed
Y. Xu, L. Wang, G. Buttice, P. K. Sengupta, B. D. Smith, *J Biol Chem* **279**, 41319 (Oct 1, 2004).	PCR
N. Yang, L. Zhang, H. H. Kazazian, Jr., *Nucleic Acids Res* **33**, e57 (2005).	PCR
S. Yang, N. B. Hecht, *FEBS Lett* **576**, 221 (Oct 8, 2004).	Annealed
Z. Yang *et al.*, *Mol Cell Biol* **25**, 4062 (May, 2005).	Annealed
Z. G. Yang *et al.*, *World J Gastroenterol* **11**, 498 (Jan 28, 2005).	Annealed
H. S. Yoon *et al.*, *Oncogene* (Apr 4, 2005).	Annealed
S. Y. Yoon *et al.*, *Mol Cells* **18**, 127 (Aug 31, 2004).	Annealed
J. Y. Yu, T. W. Wang, A. B. Vojtek, J. M. Parent, D. L. Turner, *Methods Enzymol* **392**, 186 (2005).	Annealed
J. Yuan, C. M. Dutton, S. P. Scully, *J Orthop Res* (May 28, 2005).	Annealed
Z. Yuan *et al.*, *Oncogene* **24**, 3657 (May 19, 2005).	Annealed
Y. Zeng, X. Cai, B. R. Cullen, *Methods Enzymol* **392**, 371 (2005).	Extension^
Y. Zeng, B. R. Cullen, *Nucleic Acids Res* **32**, 4776 (2004).	Annealed
Y. Zeng, R. Yi, B. R. Cullen, *Embo J* (Nov 25, 2004).	Extension^*
J. Zhang, E. Attar, K. Cohen, C. Crumpacker, D. Scadden, *Gene Ther* (May 5, 2005).	Annealed
G. Zhou, C. Li, L. Cai, *Am J Pathol* **165**, 2033 (Dec, 2004).	Annealed
H. Zhou, X. G. Xia, Z. Xu, *Nucleic Acids Res* **33**, e62 (2005).	Annealed

** Construction methods were not specifically detailed but were implied by reference to previous publications.*
*** The insert for cloning was constructed from several small sections that were ligated together forming a larger insert.*
^ The primer extension step was immediately followed by repeated thermocycling.
