# Supplementary material for: Design and cloning strategies for constructing shRNA expression vectors
Source: BMC Biotechnol. 2006 Jan 5;6:1. doi: 10.1186/1472-6750-6-1 (PMC1343552; doi:10.1186/1472-6750-6-1)

Additional file **2**

Diagrams for shRNA template generation via complementary annealed oligonucleotides or primer extension using Phi29 DNA polymerase

(**a**) *Complementary annealed oligos.* The coding region for each hairpin was contained within a single oligo (upper oligo: 5'-**GATCC**[G/A]$N_{(19-29)}$A**CTCGAG**A$N_{(19-29)}$[G/A/C]TTTTTTGG**A**-3') and its complementary equivalent (lower oligo: 5'-**AGCTT**CCAAAAAA[G/A/C]$N_{(19-29)}$A**CTCGAG**A$N_{(19-29)}$[G/A]**G**-3')(see **Methods** for cloning details). (**b**) *Primer extension using Phi29 DNA polymerase.* Each template oligo was similar in design to the upper oligo of the annealed oligo method (5'-GCGC**GGATCC**[G/A]$N_{(19-29)}$A**CTCGAG**A$N_{(19-29)}$[G/A/C]TTTTTTGG**AAGCTT**-3') except at the ends which were extended to encode the entire sequence of the RE sites plus an additional 5' 'seat' sequence to facilitate RE binding and digestion of the extended product. In addition to each unique template oligo, a short primer was designed to bind at the 3' end of the template oligo within the region encoding the termination signal and RE recognition site (5'-CGCG**AAGCTT**CCAAAAAA-3').

(**a**) Complementary annealed oligos

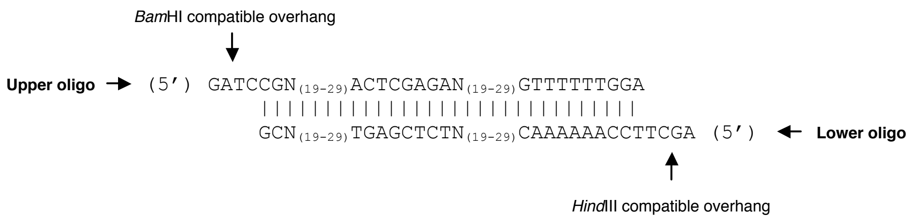

(**b**) Primer extension using Phi29 DNA polymerase

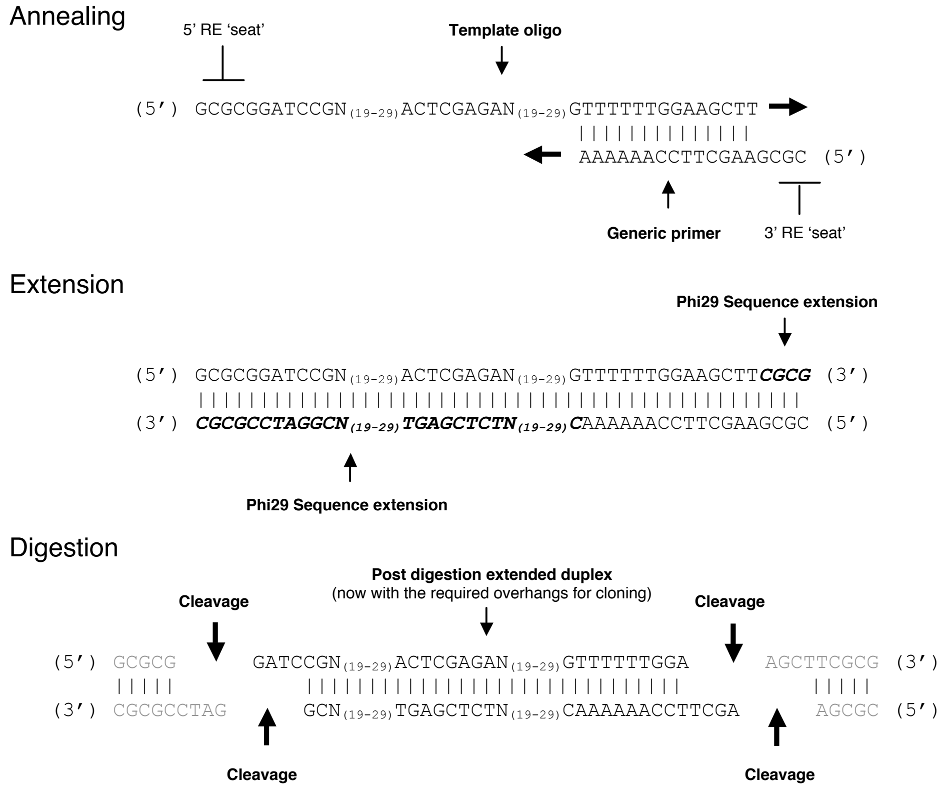

Supplement: Additional File 2 — Diagrams for shRNA template generation via complementary annealed oligonucleotides or primer extension using Phi29 DNA polymerase. Both the most common shRNA insert construction technique (complementary annealed oligos) and our proposed alternative (primer extension using Phi29) are diagrammatically represented indicating oligo alignments and the features of each oligo. [file 1472-6750-6-1-S2.pdf]
